# Energy expenditure in chow-fed female non-human primates of various weights

**DOI:** 10.1186/1743-7075-5-32

**Published:** 2008-11-17

**Authors:** Russell Rising, Maxim Signaevsky, Leonard A Rosenblum, John G Kral, Fima Lifshitz

**Affiliations:** 1 EMTAC Inc, 2219 Bath St, Santa Barbara, CA 93105, USA; 2 Department of Surgery, SUNY Downstate Medical Center, 450 Clarkson Avenue, Box 40, Brooklyn, NY 11203, USA; 3 Primate Behavior Lab, Department of Psychiatry, SUNY Downstate Medical Center, 450 Clarkson Avenue, Box 1203, Brooklyn, NY 11203, USA; 4 Pediatric Sunshine Academics, Sansum Diabetes Research Institute, 2219 Bath Street, Santa Barbara, CA 93105, USA

## Abstract

**Background:**

Until now no technology has been available to study energy metabolism in monkeys. The objective of this study was to determine daily energy expenditures (EE) and respiratory quotients (RQ) in female monkeys of various body weights and ages.

**Methods:**

16 socially reared Bonnet Macaque female monkeys [5.5 ± 1.4 kg body weight, modified BMI (length measurement from head to base of the tail) = 28.8 ± 6.7 kg/crown-rump length, m^2 ^and 11.7 ± 4.6 years] were placed in the primate Enhanced Metabolic Testing Activity Chamber (Model 3000a, EMTAC Inc. Santa Barbara, CA) for 22-hour measurements of EE (kcal/kg) and RQ (VCO_2_/VO_2_). All were fed monkey chow (4.03 kcal/g) ad-libitum under a 12/12 hour light/dark cycle. Metabolic data were corrected for differences in body weight. Results were divided into day (8-hours), dark (12 hours) and morning (2-hours) periods. Data analysis was conducted utilizing SPSS (Version 13).

**Results:**

Modified BMI negatively correlated with 22-hour energy expenditure in all monkeys (r = -0.80, p < 0.01). The large variability of daily energy intake (4.5 to 102.0 kcal/kg) necessitated division into two groups, non-eaters (< 13 kcal/kg) and eaters (> 23 kcal/kg). There were reductions (p < 0.05) in both 22-hour and dark period RQs in the "non-eaters" in comparison to those who were "eaters". Monkeys were also classified as "lean" (modified BMI < 25) or "obese" (modified BMI > 30). The obese group had lower EE (p < 0.05) during each time period and over the entire 22-hours (p < 0.05), in comparison to their lean counterparts.

**Conclusion:**

The EMTAC proved to be a valuable tool for metabolic measurements in monkeys. The accuracy and sensitivity of the instrument allowed detection of subtle metabolic changes in relation to energy intake. Moreover, there is an association between a reduction of energy expenditure and a gain in body weight.

## Background

The Enhanced Metabolic Testing Activity Chamber (EMTAC) was developed for accurate and reliable measurements of energy expenditure and respiratory quotients in humans [1-3] and rodents [4,5]. This instrument has led to important findings in regards to metabolic changes associated with various metabolic disorders in both infants [1-3,6] and rodents [4,5]. For example, infants born to overweight and obese mothers have a lower metabolic rate [1] while those with AIDS have a greater metabolic rate [3]. In rodents, the main adaptation mechanism in Sprague Dawley rats subjected to chronic suboptimal nutrition is a reduction of energy expenditure during the dark period [5]. There is a major advantage in utilizing an animal species most related to humans in regards to studying changes in energy metabolism associated with many different physiological conditions.

There have been a few attempts to utilize indirect calorimetry in non-human primates to study changes of energy expenditure in relation to energy intake. In one study, the energetic cost of bipedal versus quadrupedal walking in Japanese macaques was determined by carbon dioxide measurements while exercising on a treadmill within a respiratory chamber [7]. In another study, an indirect calorimetry system was developed and utilized to measure energy expenditure in chair-adapted primates [8]. Moreover, 24-hour energy expenditure, as determined by the Douglas bag technique, was reduced during long term caloric restriction in Rhesus monkeys (Macaca mulatta) [9]. Finally, daily energy expenditure of Rhesus Monkeys subjected to long term dietary restriction was determined by open-circuit indirect calorimetry utilizing small respiratory chambers [10]. None of these studies utilized an indirect calorimetry system that obtained continuous measurements of both oxygen and carbon dioxide concentrations while maintaining the comfort of the non-human primates.

Non-human primates are especially relevant for the study of metabolism in regards to many metabolic disorders in humans due to their genetic and physiological similarities [11,12]. The new primate EMTAC allows metabolic evaluations, including an estimate of nutrient utilization through the respiratory quotient, in monkeys subjected to different experimental protocols. We utilized this instrument to determine metabolic changes in female monkeys of various weights to determine if body weight gain is associated with changes in metabolic rate.

## Methods

### Subjects

The monkeys tested were 16 adult female Bonnet macaques (*M. radiata*) (5.5 ± 1.4 kg and 11.7 ± 4.6 years of age) socially reared in the Primate Behavioral Laboratory of the State University of New York, Downstate, Brooklyn NY. Their modified BMI [13] ranged from 20.9 to 41.6 kg/crown-rump length, m^2 ^with an average of 28.8 ± 6.7. The length measurement utilized for calculation of modified BMI is the distance from the crown of the head to the base of the tail. Their body surface area [(body weight; kg ^0.6046^)* (Length from head to anus; cm ^0.1862^) * 514; (14)] ranged from 0.25 to 0.59, with an average of 0.40 ± 0.11 m^2^. Following weaning, all monkeys were maintained on a commercial laboratory diet (Monkey Diet #5038, LabDiet Inc., Richmond, IN) containing 4.02 kcal/g and comprising of 69% carbohydrate, 18% protein and 13% fat. Normal energy intake of healthy female Bonnet Macaque monkeys in this colony at the time of this study was 40–50 kcal/kg/day depending on their body weight, age and housing situation. Their diet was occasionally supplemented with fresh fruit and/or vegetables provided once in the mid afternoon. The monkeys used in this study were housed under three different conditions. Six of them had been housed in a large group pen with three to five other female monkeys, while eight were housed in a smaller group pen along with two other female monkeys. Finally, two had been housed alone in regulation monkey cages designed for single occupancy; however these cages resided next to each other in a large room.

All monkeys had their diet and tap water provided ad-libitum. The entire monkey facility operated on a 12/12 hour light/dark cycle with the lights off at 7:00 PM and on the next day at 7:00 AM. This study was approved by the State University of New York, Downstate Medical Center Institutional Animal Care and Use Committee.

### Enhanced Metabolic Testing Activity Chamber for monkeys

**Figure 1:**
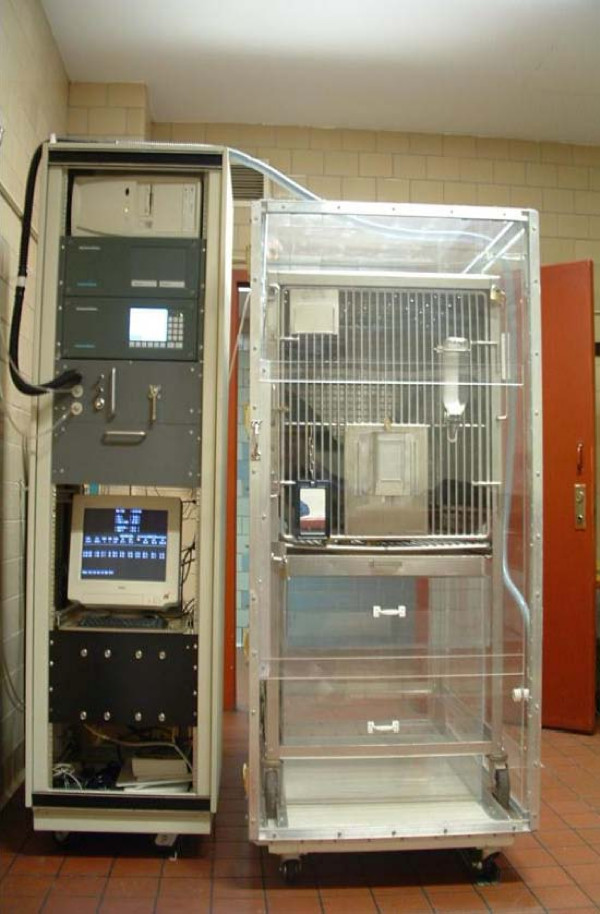
**Monkey EMTAC showing the instrument rack to the left side of the Plexiglass enclosure.** The Plexiglass enclosure is shown with the monkey metabolic cage and phantoms inside.

For this study the EMTAC was retrofitted for use in monkeys (Figure 1). This involved constructing a new Plexiglas enclosure (122 × 86 × 152 cm) with a volume of 1600 liters thus allowing for a monkey metabolic cage to be rolled inside (Figure 1). Two phantoms were placed under the cage (230 liters total) during metabolic measurements in order to reduce the internal volume to 1370 liters. This was necessary to maintain the accuracy of metabolic measurements while still maintaining the airflow though the enclosure at 35 liters/minute. A portable instrument rack housed the oxygen and carbon dioxide analyzers, air flow meter, barometric pressure, temperature and humidity sensors and computer equipment. Room air was circulated through the enclosure using inline fans (Micronel USA, Vista CA) and exhausted to the outside via the building ventilation system. Oxygen and carbon dioxide concentrations, flow rate, temperature, barometric pressure and humidity were measured continuously on the exhaust side of the system. Oxygen and carbon dioxide concentrations were determined using a two-channel Uris 14 carbon dioxide detector (ABB Automation, Houston TX) and two single channel Magnos 16 oxygen detectors (ABB Automation, Houston TX). They were configured in order to provide simultaneous measurements of both room reference air and sample air within the enclosure. This allowed for continuous correction of changes in oxygen and carbon dioxide concentrations within the room. The analyzers were sensitive to 0.001% allowing for measurements of oxygen and carbon dioxide concentrations without the need for large decreases in oxygen or increases in carbon dioxide within the enclosure. Air flow rate through the enclosure was continuously measured by a turbine transducer (Interface Associates, Laguna Nigeul, CA). Barometric pressure (mm Hg), temperature (C) and relative humidity (%) were measured using probes within the exhaust line (Omega Engineering, Stanford CT). This data were utilized to correct the airflow rate to standard temperature-pressure (STPD) conditions. Temperature and humidity within the laboratory were maintained at 24 degrees C and 30%, respectively. Data acquisition and processing was achieved utilizing an analog/digital converter (Data Translation Inc, Marlboro MA) connected to an IBM computer (IBM Inc., White Plains, NY).

### Instrument validation

The integrity of the system was tested by first conducting a 22-hour combustion test utilizing instrument grade propane (99% purity, Instrumentation Laboratory, Lexington MA). The instrument was considered accurate and precise since the respiratory quotient of the propane being combusted was maintained between 0.58 and 0.60 (0.58 ± 0.08) during the entire 22-hour test [15]. Furthermore, three healthy female monkeys not related to this study (7.2 ± 0.1 years, 4.0 ± 0.6 kg and modified BMI = 23.7 ± 3.9) had two replicate tests, three days apart. The data were in close agreement for 22-hour EE (59.3 ± 7.0 vs. 59.7 ± 5.0 kcal/kg/d) according to paired t-tests (p = 0.93). Moreover, all monkeys tolerated the length of time in the EMTAC without any apparent ill effects as verified by the staff veterinarian.

### Metabolic measurements

Prior to each metabolic measurement the EMTAC was calibrated with standard gases with known certified concentrations of oxygen and carbon dioxide (Medical Gases Inc., Westbury NY). The results from the EMTAC had to be within one percent of the concentration of oxygen and carbon dioxide of the certified calibration gases. This is an acceptable accuracy for metabolic tests using indirect calorimetry [15]. After all calibrations were complete, the monkey being studied was retrieved from their respective housing utilizing a monkey transport cage. Once in the laboratory the monkey was weighed, placed into the metabolic cage, and the whole apparatus rolled into the EMTAC Plexiglas enclosure for metabolic measurements. Only the monkey being tested within the EMTAC occupied the laboratory. Other than the EMTAC, no other equipment or personnel were allowed in the laboratory during metabolic measurements.

Metabolic measurements were performed for 22-hours from 11:00 AM till 9:00 AM the following day. Two hours were allowed for cleaning of the enclosure and instrument calibrations. Energy expenditure (kcal/min) and the respiratory quotient (VCO_2_/VO_2_) were continuously calculated during metabolic testing from the oxygen and carbon dioxide concentrations within the enclosure according to the method of Jequier [16]. The data were summarized every five minutes as described previously [2,3,6]. The computer system collected data at a rate of 600 times per minute. The light/dark cycle was maintained within the lab at 12/12 hours, respectively, with the lights off (7:00 PM) and on (7:00 AM) during similar times as with the rest of the monkey facility. After completion of the metabolic test the monkey was removed from the metabolic cage and food consumption during the preceding 22-hours was recorded. Water was provided ad-libitum during metabolic testing using water bottles attached to the metabolic cage.

### Calculations

To correct the continuous metabolic data for differences in body weight, each five minute data summary period (kcal/min) was divided by kg body weight and expressed as kcal/min/kg. Next, the corrected continuous metabolic data for each female monkey were than divided into three separate periods consisting of eight, 12 and two hours each, respectively. The light period ranged from the time the metabolic test started (11:00 AM) till when the lights went off at 7:00 PM. The dark period started from the time the lights went off till they were turned back on at 7:00 AM the next day. The final two hours of the 22-hour metabolic test (7:00–9:00 AM) was defined as the morning period. The sum of the energy expenditure of all three time periods equals the 22-hour energy expenditure. The criteria used for the division of the metabolic data into the specific time periods are similar to that used for metabolic studies in infants [2] and adults [15]. Moreover, the start and completion times of the metabolic tests closely resembles those of previous studies [2,15] in humans. Energy expended (kcal) during each of these periods (light, dark and morning) were calculated by taking the mean of these five-minute data points (kcal/min) across each time period (eight, 12 and two hours, respectively) and multiplying by the number of minutes in each (day, dark and morning). The results for 22-hour EE and for each time period were divided by body weight and expressed as kcal/kg. The mean respiratory quotient (VCO_2_/VO_2_) were also calculated over the course of each time period (light, dark and morning).

### Statistical analysis

The relationships between modified BMI and 22-hour energy expenditure were determined by Partial Pearson Correlations, controlling for the effects of increasing age.

Two different analyses of the data were performed. First, the female monkeys were classified on the bases of their energy intake. Those who consumed less than 13, or greater than 23 kcal/kg/day were considered as "non-eaters" and "eaters", respectively. These limits were chosen since mean energy intake in the "eaters" group was similar (40–50 kcal/kg/day) to that found for female Bonnet Macaque monkeys within the colony. The energy intake for the "non-eaters" was significantly less than that found for similar monkeys within the colony. Therefore, we utilized these differences as the basis for our sub-groups of "non-eaters" and "eaters". Differences between the two groups in regards to anthropometrics and metabolic parameters were determined by Independent t-test. Secondly, the continuous metabolic data for the monkeys were then classified as either "lean" or "obese" based on their modified BMIs being < 25 or > 30 (kg/crown-rump length, m^2^), respectively. This classification scheme was based on the human BMI criteria for obesity [17]. This led to five "lean" and six "obese" monkeys. Differences between the two groups in regards to anthropometrics and metabolic parameters were determined as described above for the "non-eaters" and "eaters". Furthermore, within each group one-way ANOVA with Bonferroni post hoc tests were utilized to determine differences between each of the three time periods (day, dark and morning) for energy expenditure (kcal/kg) and the respective respiratory quotients.

All data were analyzed utilizing SPSS (Version 13, Chicago, IL) and expressed as Mean ± Standard Deviation unless otherwise noted.

## Results

**Figure 2:**
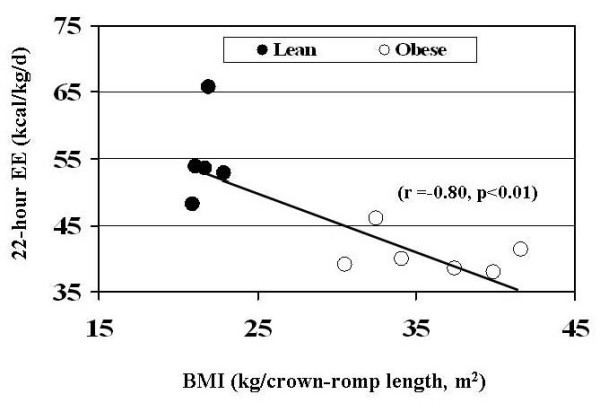
**Relationship between 22-hour energy expenditure and modified BMI for 11 female monkeys when classified as "lean" and "obese".** Correlation shown for all 11 monkeys.

Figure 2 shows the relationship between modified BMI and 22-hour energy expenditure for the monkeys that were classified into the sub-groups consisting of "lean" and "obese". No correlations between modified BMI and 22-hour energy expenditure existed separately within either the "lean" or "obese" sub-groups. However, a strong negative correlation existed when both sub-groups were considered together (Figure 2).

**Figure 3:**
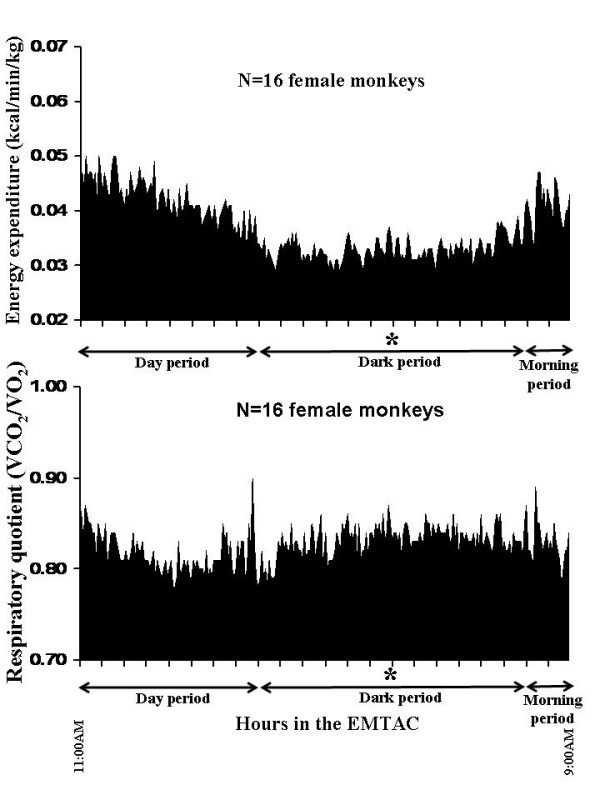
Continuous energy expenditure (kcal/min/kg, top plot) and the respiratory quotient (VCO_2_/VO_2_, bottom plot) for all 16 female monkeys (* = p < 0.05 between the day and morning periods).

The anthropometric and metabolic data, corrected for differences in body weight, for all 16 monkeys are shown in Table 1. The continuous energy expenditure and respiratory quotients over the course of the 22-hour metabolic measurement are shown in Figure 3. Energy expended was 21.6% lower during the dark period and 19.7% lower during the day and morning periods (p < 0.05). However, the respiratory quotient during the dark period was 1.6% greater (p < 0.05) than during the day time period. There were no differences in RQ between dark and morning periods.

**Table 1:** Anthropometrics and energy metabolism of the 16 monkeys in the study

Number of monkeys	16
Age (years)	11.7 ± 4.6

Body weight (kg)	5.5 ± 1.4

Modified BMI (kg/crown-rump length, m^2^)	28.8 ± 6.7

22-hour Energy intake (kcal/kg)	38.5 ± 31.6

22-hour energy expenditure (kcal/kg)	48.7 ± 8.9

Day period energy expenditure (kcal/kg)	20.1 ± 3.4

Dark period energy expenditure (kcal/kg)	23.7 ± 4.6

Morning energy expenditure (kcal/kg)	4.9 ± 1.1

22-hour respiratory quotient (VCO_2_/VO_2_)	0.83 ± 0.05

Day period respiratory quotient (VCO_2_/VO_2_)	0.82 ± 0.04

Dark period respiratory quotient (VCO_2_/VO_2_)	0.83 ± 0.07

Morning respiratory quotient (VCO_2_/VO_2_)	0.83 ± 0.07

Since there was a large variability of energy intake (38.5 ± 31.6 kcal/kg/day), with a range from 4.48 to 101.9 kcal/kg/day, the monkeys were first classified as either "non-eaters" or "eaters", based on their energy intake. No differences were observed between the "non-eaters" and "eaters" in regards to age (years), body weight (kg) or modified BMI (kg/crown-rump length, m^2^) (Table 2). However, both energy intake (kcal/kg/day) and the respiratory quotient (VCO_2_/VO_2_) were significantly lower (p < 0.05) in the "non-eaters" in comparison to those in the "eaters" group (Table 2). Moreover, no differences between the two groups were found in energy expended during either the day, dark or morning periods. However, there were reductions (p < 0.05) in both the 22-hour respiratory quotient and that during the day (p < 0.05), dark (p < 0.05) and morning (p < 0.05) periods in the "non-eaters" in comparison to the monkeys in the "eaters" group (Table 2).

**Table 2:** Anthropometrics, energy metabolism and nutrient utilization in monkeys who were "non-eaters" (< 13 kcal/kg) and "eaters" (> 23 kcal/kg) in energy intake.

	Non-eaters	Eaters
Number of monkeys	6	10
Body weight (kg)	5.4 ± 0.7	5.5 ± 1.8
Age (years)	11.8 ± 3.6	11.7 ± 5.3
Modified BMI (kg/crown rump, m^2^)	25 ± 4.2	30.3 ± 7.7
22-hour energy intake (kcal/kg)	8.0 ± 3.1	56.9 ± 11.6*
22-hour energy expenditure (kcal/kg)	47.1 ± 5.3	49.7 ± 10.6
Day period energy expenditure (kcal/kg)	20.0 ± 2.4	20.2 ± 4.0
Dark period energy expenditure (kcal/kg)	22.4 ± 2.8	24.5 ± 5.4
Morning energy expenditure (kcal/kg)	4.7 ± 0.5	5.0 ± 1.3
22-hour respiratory quotient (VCO_2_/VO_2_)	0.77 ± 0.02	0.86 ± 0.03 *
Day period respiratory quotient (VCO_2_/VO_2_)	0.80 ± 0.04	0.83 ± 0.04
Dark period respiratory quotient (VCO_2_/VO_2_)	0.75 ± 0.01	0.88 ± 0.03 *
Morning respiratory quotient (VCO_2_/VO_2_)	0.75 ± 0.02	0.87 ± 0.05 *

* = Significant (p < 0.01) between monkeys who were non-eaters and eaters

**Figure 4:**
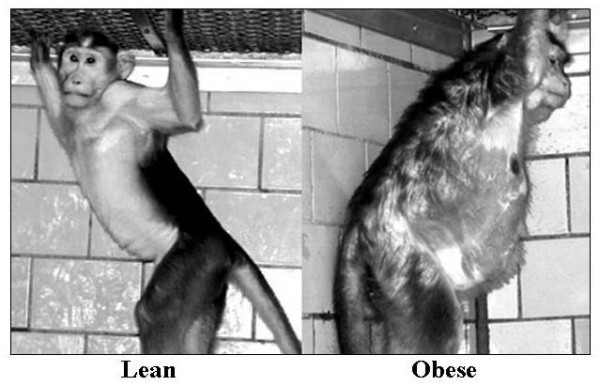
Photo of a lean (left) and an obese (right) female monkey.

When the monkeys were classified as either "lean" or "obese" (photo of each shown in Figure 4), according to their modified BMI, the obese group were heavier and older (p < 0.05). Finally, the "obese" group had lower 22-hour (p < 0.05), day (p < 0.05), dark period (p < 0.05) and morning period (p < 0.05) energy expenditures in comparison to their lean counterparts (Table 3). Finally, no changes were detected in the 22-hour respiratory quotient between the "lean" or "obese" groups. Similarly, no changes were found in the day, dark or morning period respiratory quotients between the two groups (Table 3).

**Table 3:** Anthropometrics, energy metabolism and nutrient utilization in those monkeys who were classified as "lean" or "obese" according to their modified BMI.

	Lean	Obese
Number of monkeys	5	6
Body weight (kg)	4.2 ± 0.6	6.9 ± 1.1 *
Age (years)	8.9 ± 1.6	14.7 ± 4.9 *
Modified BMI (kg/crown rump, m^2^)	21.7 ± 0.8	36.0 ± 4.3 *
22-hour energy intake (kcal/kg)	55.7 ± 45.2	23.4 ± 15.6
22-hour energy expenditure (kcal/kg)	54.8 ± 6.5	40.5 ± 3.0*
Day period energy expenditure (kcal/kg)	22.2 ± 2.1	16.9 ± 1.4*
Dark period energy expenditure (kcal/kg)	27.1 ± 3.9	19.8 ± 1.4*
Morning energy expenditure (kcal/kg)	5.5 ± 0.6	3.9 ± 0.5*
22-hour respiratory quotient (VCO_2_/VO_2_)	0.83 ± 0.05	0.82 ± 0.05
Day period respiratory quotient (VCO_2_/VO_2_)	0.81 ± 0.05	0.81 ± 0.03
Dark period respiratory quotient (VCO_2_/VO_2_)	0.83 ± 0.08	0.82 ± 0.08
Morning respiratory quotient (VCO_2_/VO_2_)	0.84 ± 0.09	0.82 ± 0.07

* = Significant (p < 0.01) between lean and obese monkeys

**Figure 5:**
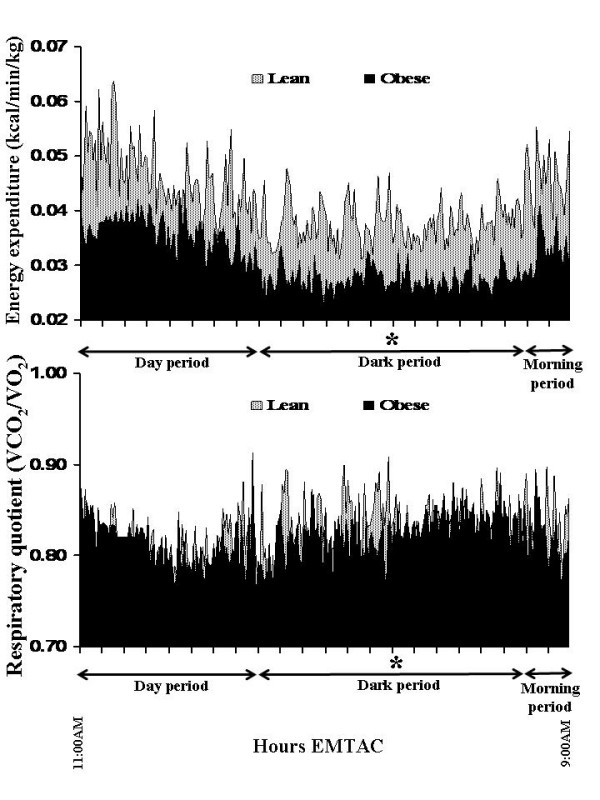
Continuous energy expenditure (kcal/min/kg, top plot) and the respiratory Quotient (VCO_2_/VO_2_, bottom plot) for the "lean" (N = 5) and "obese" (N = 6) groups over the course of the 22-hour metabolic measurement (* = p < 0.05 between the day and morning periods for both lean and obese groups).

When considering the continuous metabolic profiles, dark period energy expenditures were reduced (p < 0.05) by 18.6 and 21.8%, respectively, in both the "lean" and "obese" groups in comparison to energy expended during the day period. However, energy expenditure increased 18.6 and 16.0%, respectively, during the morning period, in comparison to the dark period, in both the "lean" and "obese" groups (Figure 5, top). In regards to the respiratory quotient, increases were observed during the dark (p < 0.05) and morning (p < 0.05) periods in both groups in comparison to the day period (Figure 5, bottom).

## Discussion

The female monkeys in this preliminary study appeared to tolerate and adapt to the 22-hour metabolic testing period without any ill effects. This is further substantiated by the reduction of continuous energy expenditure during the dark period which is suggestive of some sleeping during the time the lights were off. Moreover, the three female monkeys utilized during instrument validation had similar metabolic measurements for three consecutive days. The close agreement of these values further suggests that they quickly adapted to the metabolic chamber. Only females were utilized since the colony had a larger pool of group-housed females, providing more variance in age and weight for our study. There was a negative relationship between modified BMI [18] and 22-hour energy expenditure, corrected for differences in body size by kg body weight, in the monkeys classified according to modified BMI.

In this study the female monkeys who were classified as "non-eaters" had a lower respiratory quotient in comparison to their well fed counterparts. It is possible that their normal housing situation (single, small or large pens) might have affected their energy intake while in the EMTAC. Five of the six female monkeys in the "non-eaters" group were normally housed in a small pen with two to three others while one was housed in a large pen with a greater number of fellow monkeys. Since all of the female monkeys were tested one at a time in a primate metabolic cage within the EMTAC, it is possible that being alone, after normally being housed with other monkeys, might have affected their energy intake. This change in food intake in regards to companionship has been found in other animals and humans. For example, adult male rats ate less when fed alone versus being paired with another rodent while feeding [19]. Moreover, humans consume less food if eating alone than with either friends or family [20].

In this study the modified BMI [18] is the closest parameter utilized as an indicator of body composition in the female monkeys. This has been shown to be highly correlated to body fatness in monkeys [18,21]. Moreover, a similar relationship exists between fat-free mass and the body surface area [14]. Since there was a large range in modified BMI across the female monkeys in this study, there were classified as "lean" and "obese" according to the human criteria of obesity [17] utilizing the modified BMI. This enabled preliminary analysis into any potential metabolic differences between the lean and obese groups. Moreover, the negative relationship found between energy expenditure and the modified BMI suggests that obesity might have a metabolic cause. We found both lower 22-hour energy expenditures, as well as that during each time period, in the obese female monkeys in comparison to their lean counterparts. In adults [22] and in infants [1] a lower than average metabolic rate was found to be a predictor of body weight gain. It is possible that changes in the other components of energy expenditure (resting and sleeping) and in the level of physical activity might contribute to increasing body weight [3]. Future studies utilizing the new primate EMTAC will determine if changes in the components of energy expenditure or levels in physical activity contribute to body weight gain.

Since female monkeys were studied, it is possible that their menstrual cycle might have masked some of the metabolic changes associated with body weight gain. In a previous study in woman of childbearing age [23], there was up to a 5% difference in daily energy expenditure when passing between the lateral and follicular phases of the menstrual cycle. Reproductive hormones were not measured in our female monkeys in order to track their menstrual cycle. Since they menstruate approximately every 32 days, the changes associated with passing from the luteral to the follicular phases of the menstrual cycle might be one reason why energy expenditure of the overweight and obese groups were not even lower than that obtain in this study. A greater number of female monkeys in each group, along with an accurate assessment of their menstrual cycle status, might have led to greater reduction of energy expenditure in those classified as obese.

There have been attempts to utilize the doubly-labeled water technique to obtain accurate daily energy expenditure measurements in socially reared non-human primates. For example, the doubly labeled water technique was utilized in the study of the effects of dietary restriction on energy expenditure in Rhesus monkeys [10]. Some of the problems associated with utilizing this technique in free living animals are determining the appropriate fractionation factor to use for calculations [24], sequestration of the deuterium (^2^H_2_) in body fat [25] and changes in the total body water pool [26]. Moreover, excessive handling of animals required for injecting the dose of the labeled isotopes, along with the necessary sedation, could effect subsequent determinations of daily energy expenditure due to stress. Finally, there are associated errors with the double-labeled water method of up to 10% in obese individuals in the determination of daily energy expenditure [27]. Our new primate EMTAC eliminates the need for all the excessive handling of the animals, injections and related stress of being sedated while still providing accurate assessments of daily energy expenditure in a comfortable environment. Moreover, unlike the open-circuit indirect calorimetry system utilized in the study of Rhesus monkeys [10], our system utilizes a validated open-closed room calculation scheme [16] that accounts for minor changes in energy expenditure continuously throughout the 22-hour metabolic test [3,15]. This eliminates the need for additional time being spent in the calorimeter system for initial equilibration of respiratory exchange.

## Conclusion

We have shown that the new primate EMTAC is a sensitive and reliable instrument for metabolic measurements. This instrument might be suitable for future studies of obesity, malnutrition or any other physiological disorder that affects energy metabolism in non-human primates. Being able to accurately determine both sides of the energy balance equation (intake and expenditure) under well controlled conditions will lead to new information as to the specific metabolic changes associated with various physiological disorders, such as obesity.

## Abbreviations

ANOVA: Analysis of Variance; SD: Standard deviation; EE: Energy expenditure; RQ: Respiratory quotient; BMI: Body mass index; EMTAC: Enhanced metabolic testing activity chamber; VCO_2_: Volume of carbon dioxide; VO_2_: Volume of oxygen; Kg: Kilograms; AIDS: Acquired immune deficiency syndrome; m^2^: Square meters; C: Centigrade; STPD: Standard temperature pressure dry; Min: Minutes; ^2^H_2_: deuterium.

## Competing interests

The authors declare that they have no competing interests.

## Authors' contributions

RR has contributed to the design of the experiment and conducted the data analysis. Furthermore, he either participated in some of the actual data acquisition or supervised pediatric research fellows in this regard. He also assisted in the preparation of the small grants necessary for funding of this project. Finally, he also assisted in the writing and editing of this manuscript. FL contributed to the preparation of the manuscript and assisted with data analysis. He also edited some of the grant proposals necessary for the financial support of this study.

MS, LR and JGK were collaborators on this project and provided the expertise in regards to designing and conducting the study utilizing non-human primates. Moreover, they reviewed the data analysis and assisted with the preparation of this manuscript. All authors were involved in the final editing of this manuscript.
